# *Staphylococcus delphini* and Methicillin-Resistant *S. pseudintermedius* in Horses, Canada

**DOI:** 10.3201/eid2003.130139

**Published:** 2014-03

**Authors:** Jason W. Stull, Durda Slavić, Joyce Rousseau, J. Scott Weese

**Affiliations:** University of Guelph, Guelph, Ontario, Canada

**Keywords:** infection control, staphylococci, antimicrobial resistance, MecA protein, Staphylococcus delphini, S. pseudintermedius, methicillin-resistant S. pseudintermedius, horses, mass spectrometry, matrix-assisted laser desorption-ionization, surveillance, equine, equid, bacteria, Canada, *Suggested citation for this article*: Stull JW, Slavić D, Rousseau J, Weese JS. *Staphylococcus delphini* and methicillin-resistant *S. pseudintermedius* in horses, Canada [letter]. Emerg Infect Dis [Internet]. 2014 Mar [*date cited*]. http://dx.doi.org/10.3201/eid2003.130139

**To the Editor:** *Staphylococcus aureus* is a well-known pathogen of horses (*1*), but the role of other coagulase-positive staphylococcal species in these animals is unclear. *S. pseudintermedius* and *S. delphini*, members of the *S. intermedius* group (SIG), cause infections in some companion animals and equids (*2*), can be multidrug resistant, and could be a concern in horses. Members of SIG are difficult to differentiate by using biochemical methods and require molecular techniques for accurate species-level identification (*3*); therefore, misidentification of these pathogens could occur.

Methicillin-resistant or unusual staphylococci that are isolated at the Ontario Veterinary College Health Sciences Centre by the University of Guelph Animal Health Laboratory (AHL) routinely undergo further characterization. During 2011, the laboratory tested 5 isolates from different horses that were coagulase-positive staphylococci other than methicillin-resistant *S. aureus* (MRSA). Isolates were identified by using matrix-assisted laser desorption/ionization–time of flight (MALDI-TOF) mass spectrometry, *S. pseudintermedius* or *S. delphini* PCR (*4*), and *sod*A sequence analysis (*3*). Isolates were further characterized, as indicated, by direct repeat unit typing (*5*), pulsed-field gel electrophoresis (PFGE) (*6*), *mecA* PCR (*7*), penicillin-binding protein 2a latex agglutination test, and antimicrobial drug susceptibility testing by broth microdilution and/or disk diffusion. A search of AHL’s database was performed to identify other *S. pseudintermedius* and *S. delphini* isolates for all submissions of samples from equids during January 2011–August 2012.

Of the 5 isolates from the horses, 1 was identified as methicillin-resistant *S. pseudintermedius* (MRSP) and 4 as methicillin-susceptible *S. delphini* (Table). The MRSP isolate was classified by direct repeat unit typing as dt11a, a predominant MRSP clone in dogs in North America (*8*). In addition to β-lactams, the MRSP isolate was resistant to chloramphenicol, clindamycin, erythromycin, gentamicin, tetracycline, and trimethoprim/sulfamethoxazole and susceptible to nitrofurantoin, rifampin, streptomycin, and vancomycin.

**Table:** Results of investigation of *Staphylococcus delphini* and *S. pseudintermedius* infection in horses, Canada*

Isolate ID or source	Species	Animal age and status	Medical history	Sample source	Date sampled	Mixed infection?	Identification method†
MRSP-1	MRSP, dt11a	1 y, filly	Sinusitis	Frontal sinus surgery	2011 Aug	Yes	A
SD-1	*S. delphini* (group B)	8 y, mare	Chronic otitis externa	Ear canal swab	2011 Feb	Yes	B
SD-2	*S. delphini* (group B)	5 y, mare	*Streptococcus equi* surveillance	Nasopharyngeal wash	2011 Jun	No	B
SD-3	*S. delphini* (group A)	5 y, mare	*S. equi* surveillance	Nasopharyngeal wash	2011 Jun	No	B
SD-4	*S. delphini* (group B)	4 y, mare	*S. equi* surveillance	Nasopharyngeal wash	2011 Jul	No	B
AHLD	MRSP	4 y, mare	Cough	Respiratory tract	2011 Mar	Yes	C
	MRSP	24 y, UNK	Dermatitis (pastern)	Skin (pastern)	2011 Apr	No	C
	*S. pseudintermedius*	6 y, gelding	Chronic draining abscess	Wound	2011 May	No	C
	MRSP	8 y, mare	Nasal/sinus swelling	Upper respiratory tract	2011 Jun	Yes	C
	MRSP	19 y, mare	Previous uterine infection	Uterus	2011 Jun	Yes	C
	MRSP	11 y, gelding	Draining sores on neck	Neck wound	2011 Jul	No	C
	MRSP	4 y, mare	Wound (coronary band)	Coronary band wound	2011 Jul	Yes	C
	*S. pseudintermedius*	UNK, mare	Prebreeding examination	Uterus	2011 Aug	No	C
	*S. delphini*	3 mo, filly	Chronic pneumonia	Nasal passage	2012 Jun	No	D

*ID, identification; MRSP, methicillin-resistant *S. pseudintermedius*; AHLD, Animal Health Laboratory Database; UNK, unknown. †A, matrix-assisted laser desorption/ionization–time of flight mass spectrometry (MALDI-TOF), MRSP PCR, direct repeat unit typing, *mecA* PCR, penicillin-binding protein 2a latex agglutination test, broth microdilution; B, MALDI-TOF, *S. delphini* PCR, *sodA* sequence analysis, broth microdilution; C, standard biochemical methods, disk diffusion; D, MALDI-TOF, disk diffusion.

The 4 *S. delphini* isolates were initially identified biochemically as *S. pseudintermedius* but subsequently classified as group A (n = 1) and group B (n = 3) *S. delphini* by molecular methods (Table). One isolate (SD-4) was resistant to only erythromycin; the remaining isolates were susceptible to all tested antimicrobial drugs. PFGE showed that 2 of the *S. delphini* isolates (SD-1 and SD-2) were possibly related, with a 4-band difference. The remaining isolates were unrelated to each other and the 2 related isolates. Two of the horses (sources of isolates SD-2 and SD-3) had been recently acquired at the same auction and were sampled on the same day; however, PFGE showed that these samples were not related and came from different groups (A, B). No common epidemiologic links were identified for any of the horses.

The AHL database search identified 8 additional horses from which *S. pseudintermedius* was biochemically identified; on the basis of drug-resistance patterns, 6 (75%) of these isolates were determined to be MRSP (Table). One additional *S. delphini* isolate was identified by using MALDI-TOF. No common epidemiologic links were identified for these infections.

MRSP is an emerging pathogen in dogs and cats (*1*) but has been rarely identified in horses (*2*). The role of these bacteria in disease in horses is unclear, but given their ability to cause opportunistic infections in other species, these pathogens should not be dismissed. *S. pseudintermedius* rarely causes disease in humans (*9*), and transmission normally occurs from infected or colonized animals. Although rarely reported, infection with MRSP might be overlooked in horses; misidentification as *S. aureus* is possible if laboratories assume that coagulase-positive staphylococci from horses are *S. aureus*, and misidentification as methicillin susceptible is possible because the use of cefoxitin susceptibility and *S. aureus* breakpoints is ineffective for determination of methicillin resistance in *S. pseudintermedius* (*10*). Additionally, *S. pseudintermedius* generates coagulase-positive results by tube testing but coagulase-negative results by slide testing, which creates the potential for misidentification as coagulase-negative staphylococci*.* Given the rapid expansion of *S. pseudintermedius* infections among dogs, the potential for zoonotic transmission, and the highly resistant nature of this pathogen, ongoing surveillance is indicated in the equine population.

Recently, *S. delphini* has been divided into groups A and B (*3*). The typical hosts for group A are believed to be mustelidae (i.e., mink, ferret, badger), whereas hosts for group B remain unknown. *S. delphini* has rarely been identified in horses, but, as we observed, it may be misidentified by conventional methods. Although colonization or contamination appeared most likely in the instances we describe, these findings suggest that this opportunistic pathogen can be found in horses and might be pathogenic in certain situations.

Our findings highlight the importance of using additional identification methods (e.g., MALDI-TOF, *Staphylococcus* species–specific PCR) for differentiation of SIG members (notably *S. delphini* and *S. pseudintermedius*) to effectively document the emergence of these species in horses. In addition, these findings indicate the need to ensure proper differentiation of *S. aureus* from SIG in equine isolates, despite the historical predominance of *S. aureus*, because of the differences in methods for determination of methicillin resistance. Future studies are needed to determine prevalence trends and disease roles for these species in equids.
